# Mortality prediction among ICU inpatients based on MIMIC-III database results from the conditional medical generative adversarial network

**DOI:** 10.1016/j.heliyon.2023.e13200

**Published:** 2023-01-24

**Authors:** Wei Yang, Hong Zou, Meng Wang, Qin Zhang, Shadan Li, Hongyin Liang

**Affiliations:** a Department of Urology, The General Hospital of Western Theater Command (Chengdu Military General Hospital), Chengdu, 610083, China; b Department of General Surgery, The General Hospital of Western Theater Command (Chengdu Military General Hospital), Chengdu, 610083, China; c Department of Liver Surgery & Liver Transplantation, State Key Laboratory of Biotherapy and Cancer Center, West China Hospital, Sichuan University and Collaborative Innovation Center of Biotherapy, Chengdu 610044, Sichuan Province, China; d Department of Traditional Chinese Medicine, The General Hospital of Western Theater Command (Chengdu Military General Hospital), Chengdu, 610083, China; e Department of Gastroenterology, The 77th Army Hospital, Jiajiang, 614100, China

**Keywords:** GAN, C-med GAN, Mortality prediction, MIMIC-III, ROC-AUC

## Abstract

**Background and aims:**

Improved mortality prediction among intensive care unit (ICU) inpatients is a valuable and challenging task. Limited clinical data, especially with appropriate labels, are an important element restricting accurate predictions. Generative adversarial networks (GANs) are excellent generative models and have shown great potential for data simulation. However, there have been no relevant studies using GANs to predict mortality among ICU inpatients. In this study, we aim to evaluate the predictive performance of a variant of GAN called conditional medical GAN (c-med GAN) compared with some baseline models, including simplified acute physiology score II (SAPS II), support vector machine (SVM), and multilayer perceptron (MLP).

**Methods:**

Data from a publicly available intensive care database, the Medical Information Mart for Intensive Care III (MIMIC-III) database (v1.4), were included in this study. The area under the precision-recall curve (PR-AUC), area under the receiver operating characteristic curve (ROC-AUC), and F1 score were used to evaluate the predictive performance. In addition, the size of the dataset was artificially reduced, and the performance of the c-med GAN was compared in different size datasets.

**Results:**

The results showed that c-med GAN achieves the best PR-AUC, ROC-AUC, and F1 score compared with SAPS II, SVM, and MLP when training in the full MIMIC-III dataset. When the size of the dataset was reduced, the prediction performances of both MLP and c-med GAN were affected. However, the c-med GAN still outperformed MLP on smaller datasets and had less degradation.

**Conclusion:**

The prediction of in-hospital mortality based on the c-med GAN for ICU patients showed better performance than the baseline models. Despite some inadequacies, this model may have a promising future in clinical applications which will be explored by further research.

## 1 Introduction

Predicting mortality among intensive care unit (ICU) inpatients is critical to assessing disease severity and judging the value of new therapies, interventions, and health care initiatives. Over the past 30 years, a great deal of effort has been invested in it. Acute physiology and chronic health evaluation (APACHE) [1], simplified acute physiology score (SAPS) [2], sepsis-related organ failure assessment (SOFA) [3], and other scores have been established to predict mortality based on baseline patient characteristics. However, several validation studies conducted in different countries have shown that even the latest versions of APACHE II [4] and SAPS II [5,6] do not accurately predict actual mortality rates.

Traditional assessment models, such as SAPS II, mostly rely on logistic regression. These models impose strict restrictions on the relationship between variables and mortality risk. With the gradual progress of machine learning and deep learning methods in recent years, sophisticated network designs based on these methods have overcome the limitations of simple logistic regression models. They exhibit advantages over traditional prediction models in predicting mortality risk in ICU patients as well as in some other risk predictions. For example, the results of a study conducted by Pirracchio et al. using a super algorithm integrating multiple machine learning techniques (SICULA) showed that this method significantly outperformed traditional scores in predicting in-hospital mortality in ICU patients [7]. In the study by Wanyan et al., the heterogeneous graph model showed better results in predicting mortality in ICU patients admitted for coronavirus-19 (COVID-19) [8]. Li et al. analyzed heart failure patients admitted to the ICU using machine learning algorithms, including extreme gradient boosting (XGBoost) and least absolute shrinkage and selection operator (LASSO), and constructed a nomogram model [9]. In their study, the constructed nomogram model was able to predict the in-hospital mortality of heart failure patients admitted to the ICU, which helped to improve clinical decision-making.

With massive and accurate data, well-designed machine learning or deep learning networks can be more effective for medical risk prediction. However, the amount of clinical data, especially with appropriate labels, is limited and far from sufficient for precise predictions [10]. This is due to the following reasons [[11], [12], [13]]: (1) the diagnostic and patient labeling process is highly dependent on experienced human experts and is often very time-consuming; (2) obtaining detailed results of laboratory tests and other medical features, while becoming more feasible than ever, is still very expensive; (3) it is difficult to correlate medical data collected by different health information systems due to barriers between systems, resulting in less medical data available for scientific research; and (4) privacy concerns and regulations make it more complicated to collect and secure enough medical data. These challenges, which are distinct in health care, prevent current machine learning or deep learning models from leveraging sufficient available and high-quality labeled data to their advantage.

The generative adversarial network (GAN) is an excellent generative model in deep learning and one of the most popular research directions in artificial intelligence (AI) [14]. Yann LeCun, director of AI research at Facebook and winner of the Turing Award, praised GAN and its variations as being the most interesting ideas in the last 10 years in machine learning and deep learning in his Quora session (https://quorasessionwithyannlecun.quora.com/). Its inspiring ideas on adversarial learning have penetrated deeply into various aspects of deep learning, giving rise to a range of new research directions and various applications. At present, there are more than 500 improved variants of GAN, which have shown unexpectedly good results in data enhancement, image and medical image conversion, electronic health record data generation, biomedical data generation, and data interpolation [15]. Theoretically, GAN is potentially beneficial for predicting the mortality risk of ICU inpatients [16]. However, to the best of our knowledge, there have been no relevant studies using GAN for this problem.

The Medical Information Mart for Intensive Care III (MIMIC-III) database contains clinical data related to more than 60,000 unidentified patients in the ICU at Beth Israel Deaconess Medical Center from 2001 to 2012 [17]. It is a publicly available intensive care database maintained by the Laboratory of Computational Physiology, Massachusetts Institute of Technology (MIT). The database contains virtually all electronic patient record data that can be collected, including demographics, vital signs, test results, exam findings, operations, and medication use. It can be used for analytical studies, including epidemiology, clinical decision planning, and electronic tool development.

In this study, we constructed an improved GAN to enable its utilization in the prediction of mortality risk in ICU inpatients. Its predictive efficiency was evaluated by utilizing the MIMIC-III database.

## 2 Methods

### 2.1 Data collection & preprocessing

Data from the MIMIC-III v1.4 database for 38,597 adult patients (15 years of age and older) between 2001 and 2012, were collected and included in the study.

After a localized deployment of the MIMIC-III v1.4 database, the PostgreSQL Database Management System v14.2 software (The PostgreSQL Global Development Group & Regents of the University of California) was used to manage and extract data. Extracted data included features, consisting of patient demographics, diagnosis, vital signs, test results, treatment, other relevant information, and death labels. Relevant measures, including fluid balance and severity assessment, were also constructed based on official MIMIC database documentation (https://github.com/MIT-LCP/mimic-code/tree/main/mimic-iii) [18]. Ultimately, a dataset including 136 features as well as death labels was established, referring to a benchmarking study on the MIMIC-III database [19]. For repeated admissions or repeated ICU admissions of the same patient, only data from his or her first ICU admission were included.

Previously developed and validated data preprocessing protocols were used in this study [20]. These include imputation of missing values, hot-coding of categorical variables as numeric dummy variables, data harmonization and aggregation across multiple data tables, and normalization to facilitate cross-feature distance calculations.

The dataset was divided into three sizes: (1) the full dataset (2) the small dataset, consisting of approximately 10% of patients randomly selected; and (3) the medium dataset, consisting of approximately 50% of patients randomly selected. The data in both the small and medium datasets were resampled 5 times and mean values were calculated.

### 2.2 Ethical issues

The study was approved by the Institutional Review Board of General Hospital of Western Theater Command. Because the study did not affect clinical treatment and care and all protected health information was deidentified, the requirement for individual patient consent was waived.

## 3 Prediction models

### 3.1 SAPS II score

In the study on SAPS II scores, the investigators proposed a parsimonious formula for directly estimating patient mortality using SAPS II scores [6]: $\log\left[\frac{\text{pr}\left(\text{death}\right)}{1-\text{pr}\left(\text{death}\right)}\right]=-7.7631+0.0737*\text{SAPSII}+0.9971*\log\left(1+\text{SAPSII}\right)$. Using this formula, we estimated the inpatient mortality directly.

### 3.2 Support vector machine

Support vector machines (SVMs) are a class of generalized linear classifiers that perform binary classification of data in a supervised learning manner. SVM is one of the common kernel learning methods, for which the decision boundary is the maximum-margin hyperplane for learning samples. In this study, feature variables in the training set were utilized to train the SVM, and feature variables in the validation set were utilized to predict mortality.

### 3.3 Multilayer perceptron

**Fig. 1:**
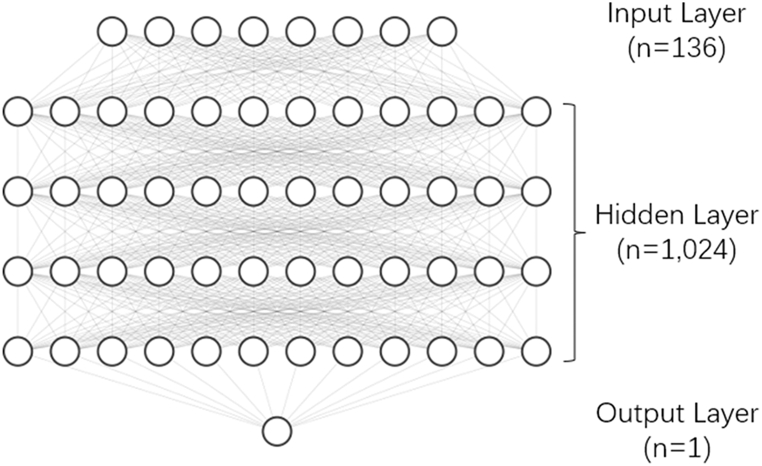
Multilayer perceptron.

In this study, a standard multilayer perceptron (MLP) model was used (as shown in Fig. 1). This MLP had 5 layers. The number of nodes in the input layer was the number of features (136), the number of nodes in the output layer was 1, and the number of nodes in the remaining hidden layers was 1,024. The loss function used was binary cross-entropy (BCEloss).

### 3.4 Conditional medical GAN

**Fig. 2:**
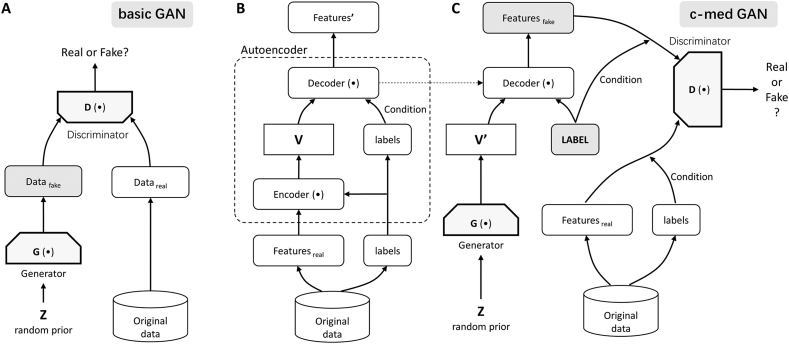
GAN and conditional medical GAN (c-med GAN). (A) Basic GAN. The generator (G) network generates fake data from the random variable Z. The discriminator (D) network distinguishes between fake data and real data. In constant iterations, G is able to generate fake data that even D cannot distinguish from the real data. (B) Autoencoder network in the c-med GAN. The features and labels were extracted separately from the original data and put into the encoder module to produce the intermediate vector V. The intermediate vector V and labels were then put into the decoder module for training. The goal of training is to reproduce the output features identical to the input features. (C) Adversarial networks in the c-med GAN. G first generated variables V', with the same dimensionality as the intermediate variables V in the autoencoder, from the random variable Z. Then, V' was put into the trained decoder module together with the given labels to generate the fake features. Eventually, the given labels and fake features were put into D along with the labels and features of the real data to discriminate their authenticity. The goal of training is to enable G to generate fake data with labels that D cannot distinguish from the real data.

The basic GAN (shown in Fig. 2A) consists of a generator (G) network and a discriminator (D) network. Through continuous adversarial learning, the generator can produce fake data that even the discriminator cannot distinguish from the real data. GANs were first applied in the field of image generation [14].

However, basic GAN does not work satisfactorily for the processing of discrete data. Cui et al. improved it by using an autoencoder network in a generator and called it medical GAN (medGAN) [21]. In medGAN, an autoencoder network with an encoder module and a decoder module is first trained, and then the decoder module is substituted into the GAN network for the next training step. MedGAN can be used to better generate medical record data with high-dimensional multilabel discrete variables (binary and count variables such as diagnoses, medications, and procedure codes).

Conditional GAN (CGAN) is also a variant of GAN [22]. In CGAN, by adding conditional constraints (labels) to the generator and discriminator, it is possible to generate fake data with specific characteristics as in real data. In this study, we combined the advantages of medGAN and CGAN to construct a conditional medical GAN (c-med GAN) (as shown in Fig. 2B and C). With the c-med GAN, we can generate fake data with labels similar to real data, which has the potential to improve the prediction of mortality in ICU patients. The generated data were added to the real data at a ratio of 1:1 and then trained in another MLP to obtain the prediction results in this study.

In the autoencoder network of the c-med GAN, the goal of training is to make it possible to reproduce the output features identical to the input features. In the adversarial network, the goal of training is to enable the generator to generate fake data with labels that the discriminator cannot distinguish from the real data. The loss functions of the autoencoder, generator, and discriminator are referred to a variant of medGAN, which is called medWGAN [23].

## 4 Model optimization and evaluation

**Fig. 3:**
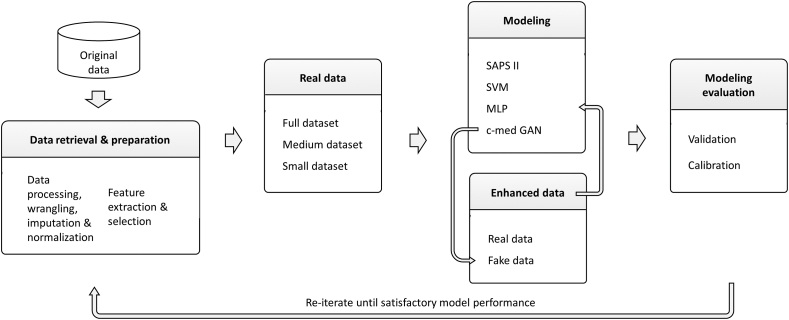
The workflow the of pipeline used in this study.

The development and testing pipeline is shown in Fig. 3. The dataset underwent rigorous training and a 5-fold grid search cross-validation process to find the optimized hyperparameters and then the optimized model selection. The bootstrapping method was used to find the 95% confidence intervals (CI) of performance metrics.

### 4.1 Receiver operating characteristic (ROC) curve

The ROC curve is a coordinate graphical analysis tool. ROC analysis can provide objective and neutral advice regardless of cost/benefit in decision-making. The value of the area under the ROC curve (ROC-AUC) is the size of the area under the ROC curve. Generally, the value of ROC-AUC is between 0.5 and 1.0, and a larger ROC-AUC represents better performance. However, the evaluation of model performance with AUC-ROC is likely to be susceptible to class imbalance.

### 4.2 Precision-recall (PR) curve

The PR curve is a curve made with the variables precision and recall, where recall is the horizontal coordinate and precision is the vertical coordinate. The area under the PR curve (AUC-PR), which is also called average precision, is also calculated by the area under the PR curve. In the case of highly skewed datasets, AUC-PR is more effective than AUC-ROC in reflecting the performance of the classifier.

### 4.3 F1 score

The F1 score is a statistical measure used in binary classification tasks. The F1 score can be considered a kind of summed average of model precision and recall, its maximum value is 1 and minimum value is 0. The F1 score is also positively correlated with the predictive performance of the model. The F1 score is usually less affected by class imbalance.

### 4.4 Calibration curve

The consistency between the predicted and actual results was assessed with flexible calibration curves. The calibration curve is a visualization of the results of the Hosmer‒Lemeshow goodness-of-fit test [24]. The intercept of the calibration curve and standard curve reflects the prediction confidence of the mode.

## 5 Experimental environment and statistical analysis

This study was conducted on a computer with an NVIDIA(R) RTX(R) 2060 GPU and Intel(R) Xeon(R) CPU E−2224G processor. PostgreSQL Database Management System v14.1 (The PostgreSQL Global Development Group & Regents of the University of California, USA) and Python 4.1.0 (Python Software Foundation, Wilmington, DE, USA) were used for data extraction and preprocessing, model development and validation, visualization and statistical analysis. SVM, MLP, and c-med GAN were implemented through the Sklearn and PyTorch packages in Python. Descriptive statistics were used to describe patient characteristics and are expressed as the means ± standard deviations (SDs) or absolute numbers (proportions) as appropriate. P < 0.05 was considered statistically significant.

## 6 Results

**Fig. 4:**
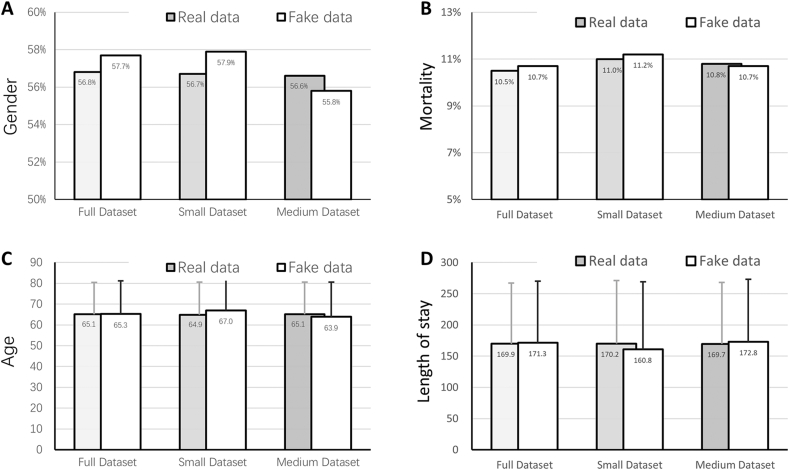
Comparison of some basic characters between real and fake data in datasets. The fake data were generated by thec-med GAN. The small and medium datasets included 10% and 50% of patients in the full dataset, respectively.

The comparison of some basic characteristics between datasets is shown in Fig. 4(A-D). There was no significant difference in in-hospital mortality (full dataset, 10.5%; small dataset, 11.0%; medium dataset 10.8%; P > 0.05), gender (full dataset, male 56.8%; small dataset, male 56.7%; medium dataset, male 56.6%; P > 0.05), age (full dataset, 65.1 ± 15.3; small dataset, 64.9 ± 15.7; medium dataset 65.1 ± 15.5; P > 0.05), or length of stay (LOS) (full dataset, 169.9 ± 97.3; small dataset, 170.2 ± 100.9; medium dataset, 169.7 ± 98.4; P > 0.05) among the three datasets. The fake data generated by the c-med GAN are also shown in Fig. 4. In small datasets, the difference between fake data and real data is larger than that of the medium dataset and full dataset, but there is no significant difference (P > 0.05).

**Fig. 5:**
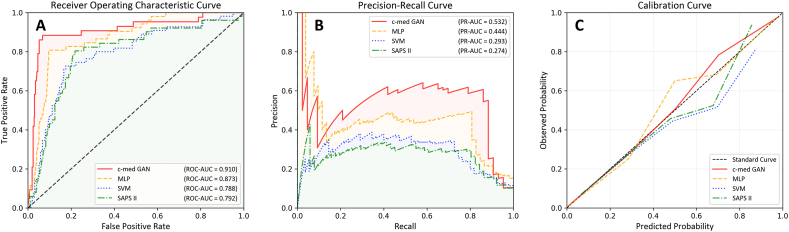
Comparison of the predictive performance between different models. A. Receiver operating characteristic curve, B. precision-recall curve, C. calibration curve.

We compared the performance of SAPS II, SVM, MLP, and c-med GAN on in-hospital mortality prediction in the full dataset. The c-med GAN achieved the best performance. The results in Table 1 and Fig. 5(A-C) show that the c-med GAN obtained the highest PR-AUC (0.532; 95% CI: 0.494–0.579), ROC-AUC (0.910; 95% CI: 0.881–0.937) and F1 score (0.551; 95% CI: 0.509–0.594). Although the F1 score of the SVM (0.429 [95% CI: 0.368–0.486]) was higher than that of the SAPS II (0.376 [95% CI: 0.366–0.486]), the ROC-AUC (0.788 [95% CI: 0.766–0.811] vs. 0.792 [95% CI: 0.771–0.808]) and PR-AUC (0.293 [95% CI: 0.272–0.326] vs. 0.274 [95% CI: 0.267–0.308]) of both were similar. Compared to SAPS II and SVM, the c-med GAN obtained an approximately 15% improvement in the ROC-AUC and an approximately 80% improvement in the PR-AUC. Even compared to the better performing MLP, the ROC-AUC of the c-med GAN was improved by 4.6,% and the PR-AUC was improved by 20.5%.

**Table 1:** Comparison of multiple mortality prediction models.

	PR-AUC1	ROC-AUC2	F1 score	precision score	recall score
SAPS II3	0.274 (95% CI4: 0.267–0.308)	0.792 (95% CI: 0.771–0.808)	0.376 (95% CI: 0.307–0.439)	0.754 (95% CI: 0.750–0.758)	0.256 (95% CI: 0.252–0.260)
Support vector machines	0.293 (95% CI: 0.272–0.326)	0.788 (95% CI: 0.766–0.811)	0.431 (95% CI: 0.368–0.486)	0.824 (95% CI: 0.820–0.828)	0.325 (95% CI: 0.320–0.330)
Multilayer perceptron	0.444 (95% CI: 0.401–0.472)	0.873 (95% CI: 0.858–0.903)	0.460 (95% CI: 0.398–0.516)	0.821 (95% CI: 0.817–0.825)	0.337 (95% CI: 0.332–0.341)
c-med GAN4	0.532 (95% CI: 0.494–0.579)	0.910 (95% CI: 0.881–0.937)	0.551 (95% CI: 0.509–0.594)	0.874 (95% CI: 0.871–0.878)	0.441 (95% CI: 0.436–0.445)

Abbreviations: 1PR-AUC: area under the precision-recall curve; 2ROC-AUC: area under the receiver operating characteristic curve; 3SAPS II: simplified acute physiology score II; 4CI: confidence interval; 5c-med GAN: conditional medical generative adversarial networks.

**Fig. 6:**
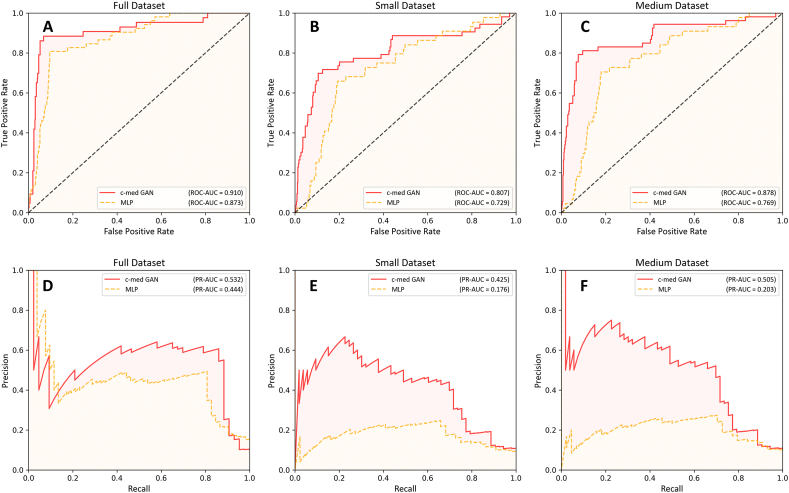
Comparison between the multilayer perceptron and c-med GAN in the different size datasets. A. receiver operating characteristic curve in the full dataset, B. receiver operating characteristic curve in the small dataset, C. receiver operating characteristic curve in the medium dataset. D. precision-recall curve in the full dataset, E. precision-recall curve in the small dataset, F. precision-recall curve in the medium dataset.

We further compared the performance of the MLP and c-medGAN on different datasets (as shown in Table 2 and Fig. 6(A-F)). In the small dataset, the predictive performance of both models was affected. In the small dataset, the PR-AUC for MLP decreased from 0.444 (95% CI: 0.401–0.472) to 0.176 (95% CI: 0.152–0.196), decreasing by 60.4%, and the PR-AUC for c-med GAN decreased from 0.532 (95% CI: 0.494–0.579) to 0.425 (95% CI: 0.399–0.448), decreasing by 20.1%. The F1 score for MLP was reduced from 0.460 (95% CI: 0.398–0.516) to 0.311 (95% CI: 0.198–0.395), decreasing by 32.4%, and the F1 score for c-med GAN was decreased from 0.551 (95% CI: 0.509–0.594) to 0.387 (95% CI: 0.269–0.503), decreasing by 29.8%; ROC-AUC for MLP was decreased from 0.873 (95% CI: 0.858–0.903) to 0.726 (95% CI: 0.628–0.822), decreasing by 16.8%, and ROC-AUC for c-med GAN was reduced from 0.910 (95% CI: 0.881–0.937) to 0.796 (95% CI: 0.679–0.913), decreasing by 12.5%. In the medium dataset, the PR-AUC of the c-med GAN decreased by 5.1%, the F1 score decreased by 13.1%, the ROC-AUC decreased by 4.6%, the PR-AUC of MLP decreased by 54.3%, the F1 score decreased by 13.1%, and the ROC-AUC decreased by 12.1%.

**Table 2:** Comparison of mortality prediction between datasets.

		PR-AUC1	ROC-AUC2	F1 score
Multilayer perceptron	Full dataset	0.444 (95% CI3: 0.401–0.472)	0.873 (95% CI: 0.858–0.903)	0.460 (95% CI: 0.398–0.516)
	Small dataset	0.176 (95% CI: 0.152–0.196)	0.726 (95% CI: 0.628–0.822)	0.311 (95% CI: 0.198–0.395)
	Medium dataset	0.203 (95% CI: 0.181–0.224)	0.767 (95% CI: 0.688–0.847)	0.381 (95% CI: 0.317–0.439)
c-med GAN4	Full dataset	0.532 (95% CI: 0.494–0.579)	0.910 (95% CI: 0.881–0.937)	0.551 (95% CI: 0.509–0.594)
	Small dataset	0.425 (95% CI: 0.399–0.448)	0.796 (95% CI: 0.679–0.913)	0.387 (95% CI: 0.269–0.503)
	Medium dataset	0.505 (95% CI: 0.478–0.526)	0.868 (95% CI: 0.807–0.929)	0.479 (95% CI: 0.417–0.539)

Abbreviations: 1PR-AUC: area under the precision-recall curve; 2ROC-AUC: area under the receiver operating characteristic curve; 3CI: confidence interval; 4c-med GAN: conditional medical generative adversarial networks.

## 7 Discussion

Our results show that c-med GAN can effectively improve the prediction of mortality for ICU patients compared to SAPS II, SVM, and MLP. When the size of the dataset was reduced, the prediction performance of both MLP and c-med GAN was affected. However, the c-med GAN still outperformed MLP in smaller datasets for mortality prediction and had less degradation.

The recent successes and developments in deep learning are revolutionizing many domains, such as computer vision (CV) and natural language processing (NLP) thereby bringing significant innovations and applicable solutions [25]. GAN is an excellent generative model that has been shown to work well in many fields. In medical informatics, GAN has demonstrated good performance in data simulation. The medGAN adopted in our study can overcome the limitations of the basic GAN by using autoencoders to generate medical record data with high-dimensional multilabel discrete variables (binary and count variables such as diagnoses, medications, and procedure codes) [21]. Baowaly et al. optimized medGAN, using Wasserstein GAN with gradient penalty (medWGAN) and boundary-seeking GAN (medBGAN), which can make synthetic medical data more realistic [23]. Yoon et al. used external datasets from related but different hospitals as auxiliary datasets for the GAN thus enabling the distribution of medical data from one hospital to be better matched with the distribution of medical data from another which in turn effectively expands the target dataset [26]. Esteban et al. used recurrent GAN (RGAN) and recurrent conditional GAN (RCGAN) to generate realistic real-valued multidimensional time series of medical data [27].

The synthetic data using GAN are mostly unlabeled or the synthetic data labels are only consistent with the original data distribution. There is no study to show the potential association of the synthetic data labels with the feature variables by GAN. For this reason, GANs often use semisupervised models to train and classify medical data after they are generated. For example, Li et al. proposed a semisupervised learning framework for rare disease detection using GAN [28]. This approach, which uses a large amount of unlabeled data, achieves the best results in terms of recall scores compared to the baseline techniques. Che et al. used two longitudinal real medical datasets of heart failure and diabetes to study the effect of GAN in generating medical data. In this study, the discriminator adopts the structure of the basic prediction model, and the generator is changed to a semisupervised learning approach [29]. Yang et al. proposed another semisupervised approach related to GAN to support medical decision-making [30]. In their study, GAN generates synthetic data by using the marker set as input. Both the extended marker set and the synthetic set were used as training sets to classify the stroke dataset based on the stroke dataset collected from the health-IoT platform.

In this study, we took advantage of medGAN, combined with CGAN, and added labels to the autoencoder, generator, and discriminator to effectively generate data with labels, thereby improving the accuracy of mortality prediction for ICU patients. The learning of data with labels will greatly reduce the cost of computation and time compared to using data without labels [31].

Our work can also be seen as a contribution to data augmentation. Therefore, in this study, we artificially reduced the data size in the database. The reduction in data size caused a decline in the predictive performance, but the c-med GAN could compensate for this decline. This showed the good effect of the model on the augmentation of a smaller-size dataset. Since the difficulties of collecting and privacy concerns of medical data limit the scale of medical data, c-med GAN has potential for applications.

The basic GAN has problems in terms of training instability, gradient disappearance, and pattern collapse. In wGAN-GP, the discriminator is replaced with a fit to the Wasserstein distance, and a gradient penalty is added [32]. The Wasserstein distance is a fine method to measure the distance of two distributions with gradient smoothing [33]. Thus wGAN-GP is a better solution to the training instability problem of the basic GAN. Therefore, similar to Baowaly's study [23], we used wGAN-GP instead of basic GAN which makes the training more stable.

In basic GAN, the optimal discriminator can be equivalently transformed to the Jensen–Shannon (JS) divergence between the true distribution and the generated distribution [14]. One important reason for medGAN adopting an autoencoder structure is that JS divergence is not smooth when dealing with discrete variables. This results in the discriminator not being able to pass the gradient to the generator, which makes the gradient update problematic. In wGAN-GP, the optimal discriminator can be equivalently transformed to the Wasserstein distance between the true distribution and the generated distribution [34]. The Wasserstein distance can overcome the above problem of JS divergence very well. The output layer of the generator and the real samples can be fed together into the discriminator, and the gradient can be directly updated by backpropagation in wGAN-GP [35]. In theory, it is possible to achieve similar results as medGAN for discrete variable processing by directly applying the wGAN-GP. In this study, we nevertheless adopted an autoencoder structure similar to medGAN, whose purpose is not only to increase the effect of processing discrete variables but also, more importantly, to add conditional constraints to the autoencoder so that the generator can generate medical data with labels more efficiently.

In this study, we used in-hospital death as an input label to generate similar medical data for patients with in-hospital deaths and patients with non-in-hospital deaths. By changing the input labels, the c-med GAN can be made to generate data with different labels, which makes the c-med GAN have better naturalization performance and may be utilized in other risk prediction models. This is, however, subject to further experimental validation.

There are still a few limitations in this study. First, the setup of our dataset was relatively simple, and we included as much data as could be collected in the MIMIC-III database. These may include some aberrant data prior to the patient's death, which is often significantly anomalous from normal data. In practice. These data are difficult to obtain, or they are obtained when the patient has an irreversible trend toward death. This limits the scalability of the results. Second, for the interpretability of the results, we adopted only simple models, such as MLP, and did not use complex models, such as convolutional neural networks (CNNs), recurrent neural networks (RNNs), and long short-term memory (LSTM) [36]. Hence, in this study, we were not able to demonstrate the superiority of the c-med GAN for models other than the baseline model, which slightly reduces the strength of the results. Third, significant class imbalance was present in this study. Although we used the PR-AUC and F1 scores, which were better evaluated in the case of class imbalance, it might still have an impact on the prediction effect due to class imbalance. Fourth, the efficiency of supervised models is theoretically superior to unsupervised and semisupervised learning models, but we did not have specific confirmation in this study. Finally, the lack of external validation also reduced the level of evidence for the study. These are the next steps we plan to address in our future work.

In summary, c-med GAN-based in-hospital mortality prediction for ICU patients has a better performance compared with the baseline models. Despite some inadequacies, c-med GAN may have a promising future in terms of clinical applications. This will be explored in further research.

## Author contribution statement

Wei Yang; Hong Zou: Conceived and designed the experiments; Performed the experiments.

Meng Wang: Analyzed and interpreted the data.

Qin Zhang Shadan Li: Contributed reagents, materials, analysis tools or data.

Hongyin Liang: Conceived and designed the experiments; Wrote the paper.

## Funding statement

This research did not receive any specific grant from funding agencies in the public, commercial, or not-for-profit sectors.

## Data availability statement

Data will be made available on request.

## Declaration of interest's statement

The authors declare no conflict of interest.
